# Solitary Lichen Planus Keratosis: A Potential Clinical and Dermoscopic Mimic of Malignant Skin Neoplasm

**DOI:** 10.7759/cureus.83630

**Published:** 2025-05-07

**Authors:** Lamia Mansour Billah, Soumiya Chiheb, Mohammed Oukabli, Madiha Eljazouly

**Affiliations:** 1 Dermatology and Venerology, Sheikh Khalifa University Hospital, Casablanca, MAR; 2 Dermatology, Cheikh Khalifa International University Hospital, Mohammed VI University of Health Sciences, Casablanca, MAR; 3 Pathology, Mohammed V Military Teaching Hospital, Rabat, MAR

**Keywords:** basal cell carcinoma, dermoscopy, orange areas, skin biopsy, solitary lichen planus keratosis

## Abstract

Solitary lichen planus keratosis (SLPK) is a benign cutaneous lesion, often misdiagnosed due to its clinical and dermoscopic similarities to malignant conditions like basal cell carcinoma (BCC) and melanoma. We present two case reports: an 82-year-old male with an erythematous, scaly lesion on his back, and a 66-year-old female with an itchy lesion on her thigh while undergoing chemotherapy for multiple myeloma. Dermoscopic findings included orange-erythematous backgrounds with scales, sharp borders, telangiectasias, and shiny white blotches in both patients. Histopathological analysis confirmed the diagnosis of SLPK in both cases. Although lesions often regress spontaneously, treatment options such as cryotherapy and curettage are available for symptomatic cases. Dermoscopy lacks pathognomonic features, but clues such as orange areas and scales in non-photo-exposed areas may aid in diagnosis. However, due to its potential to mimic malignant lesions, histopathological confirmation remains essential for accurate diagnosis. Clinicians should be aware of SLPK’s clinical subtypes and dermoscopic features to avoid unnecessary excisions, as it is a benign condition with no carcinomatous potential.

## Introduction

Solitary lichen planus keratosis (SLPK), also known as lichenoid keratosis (LK), lichen planus-like keratosis (LPLK), or involuting lichenoid plaque, is a benign cutaneous lesion first described in 1966 by Lumpkin and Helwig [1]. This entity is often underrecognized by clinicians, presenting with various misleading clinical appearances and different dermoscopic features [1,2]. However, SLPK appears to be relatively common in skin histopathology. The lack of pathognomonic features often leads to diagnostic uncertainty, highlighting the importance of histopathological confirmation [2,3]. We report two cases of SLPK with distinct clinical and dermoscopic presentations, both of which underscore the diagnostic challenges and the importance of clinicopathological correlation in reaching the correct diagnosis.

## Case presentation

Observation 1

**Figure 1:**
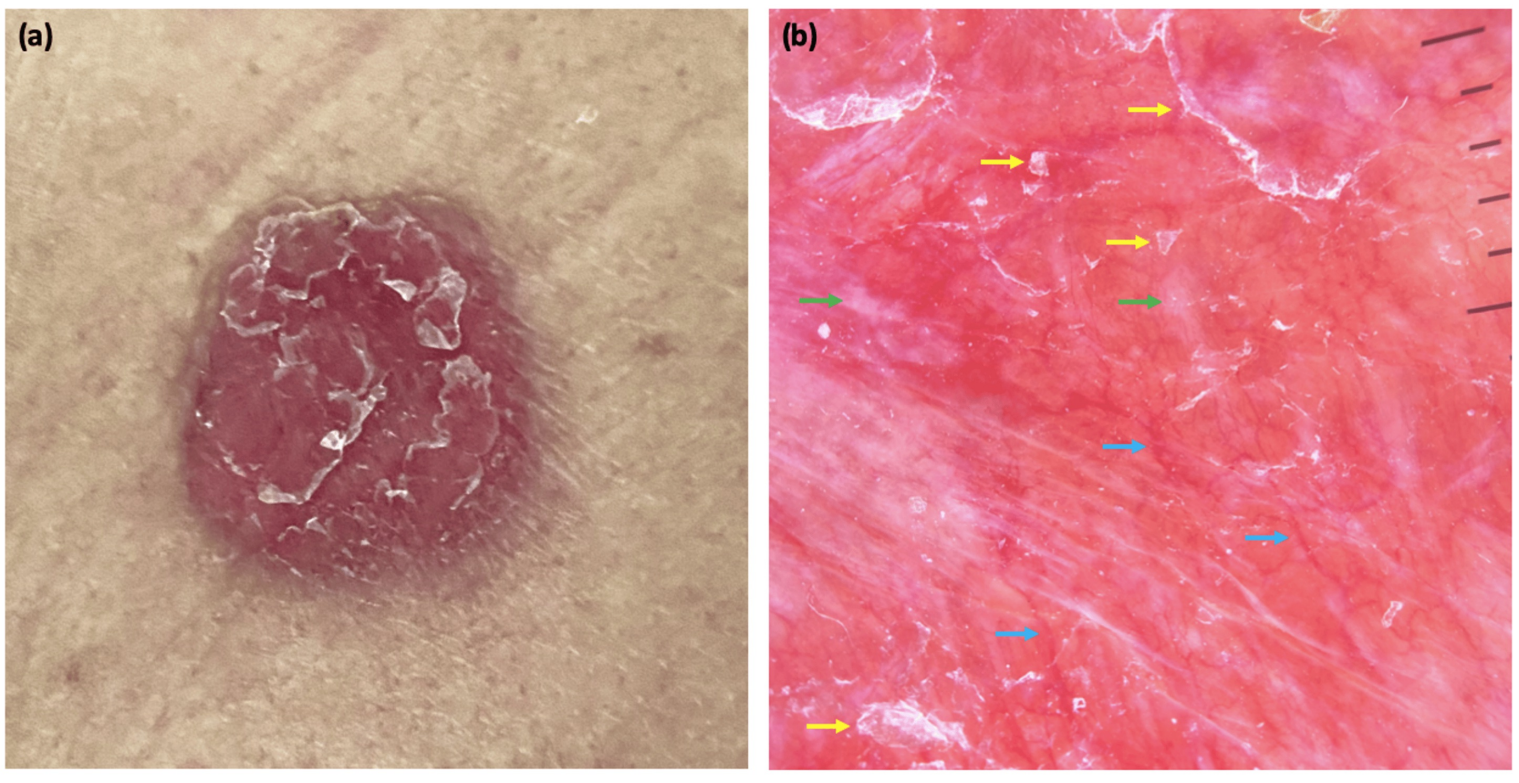
Clinical and dermoscopic features of the first patient Flat, rounded, scaly, and erythematous lesion on the mid-back (a). Dermoscopy showing telangiectatic vessels (blue arrow), scales (yellow arrow), and shiny white blotches (green arrow) on an erythematous background (b).

An 82-year-old male with no significant medical history presented with a flat, erythematous, finely scaly, rounded lesion measuring 1.3 cm in diameter on the mid-back, which had been evolving over several months. He denied any associated symptoms, drug intake, or insect bites. Dermoscopic evaluation revealed an erythematous background with scale, sharp borders, telangiectatic vessels, and shiny white blotches (Figure 1). BCC was the initial diagnosis considered. Blood tests were normal. Punch biopsy with histopathological examination confirmed the diagnosis of SLPK. Spontaneous regression of the lesion was observed over the following months.

Observation 2

**Figure 2:**
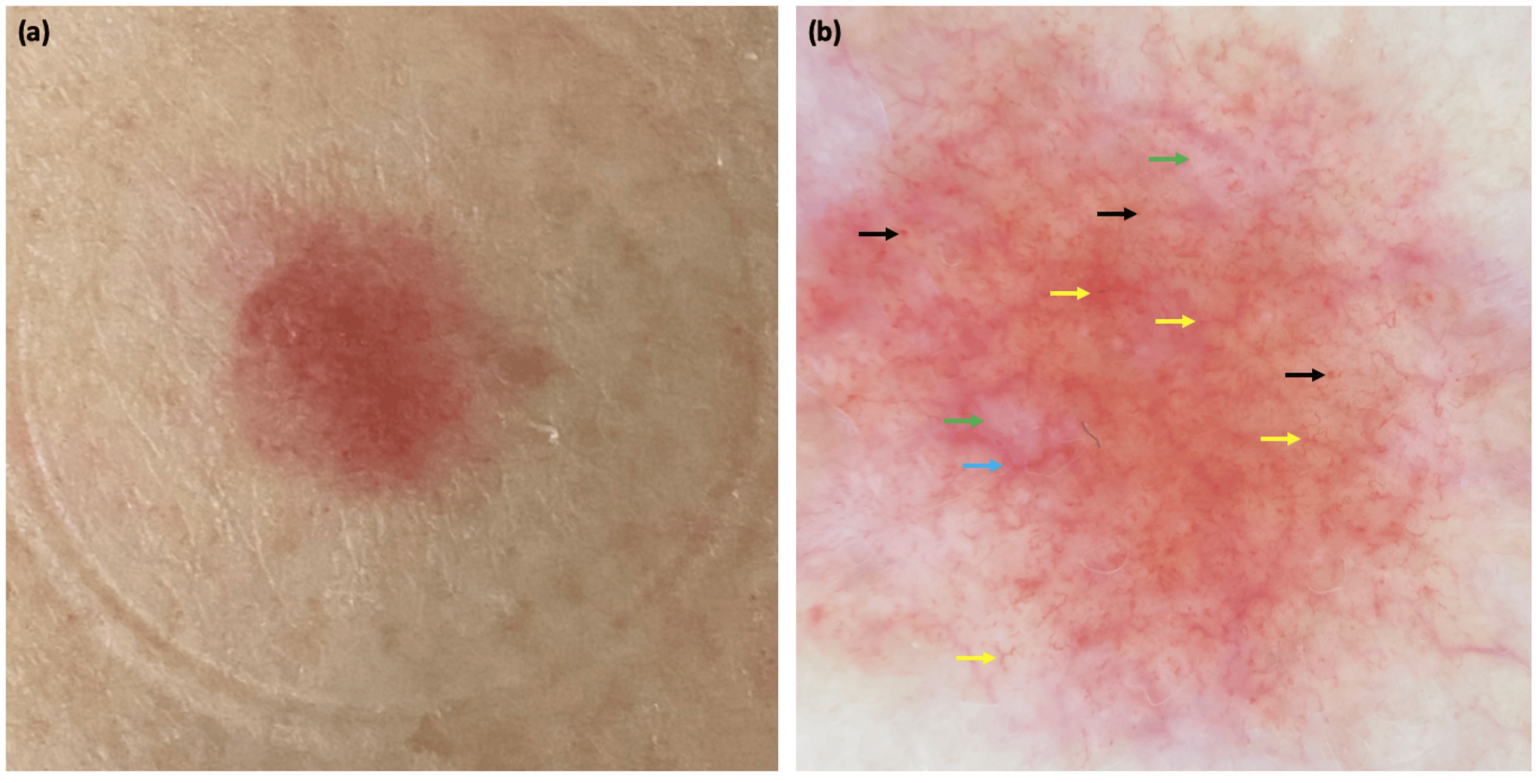
Clinical and dermoscopic features of the second patient Flat, pink-erythematous lesion on the upper thigh (a). Dermoscopic findings: red-orange background, linear vessel (yellow arrow), telangiectasia (blue arrow), dotted vessels (black arrow), and shiny white blotches (green arrow) (b).

A 66-year-old female, undergoing chemotherapy (bortezomib) for multiple myeloma, reported a two-week history of an itchy, pink-erythematous lesion on her upper thigh, measuring 8 mm. The clinical examination was unremarkable, with no fever, no palpable local lymphadenopathy, or other associated signs. Dermoscopic features included a red-orange background, linear vessels crossing each other to create a network-like pattern, telangiectasia, dotted vessels, and shiny white blotches (Figure 2).

**Figure 3:**
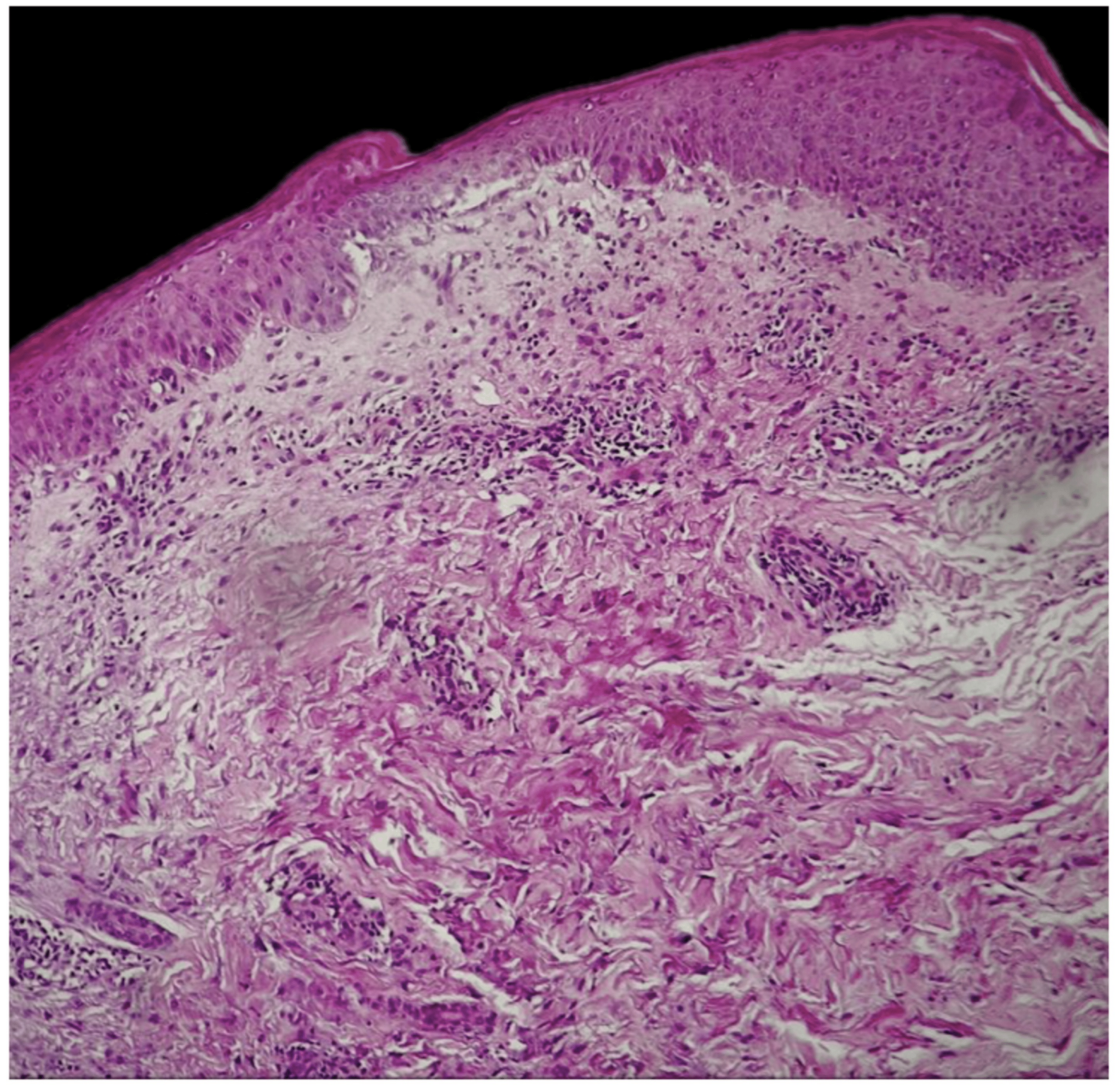
Histopathology features H&E stained at 25× original magnification: lichenoid inflammatory infiltrate, spongiform changes of the epidermis, with exocytosis of lymphoid cells. H&E: hematoxylin and eosin.

**Figure 4:**
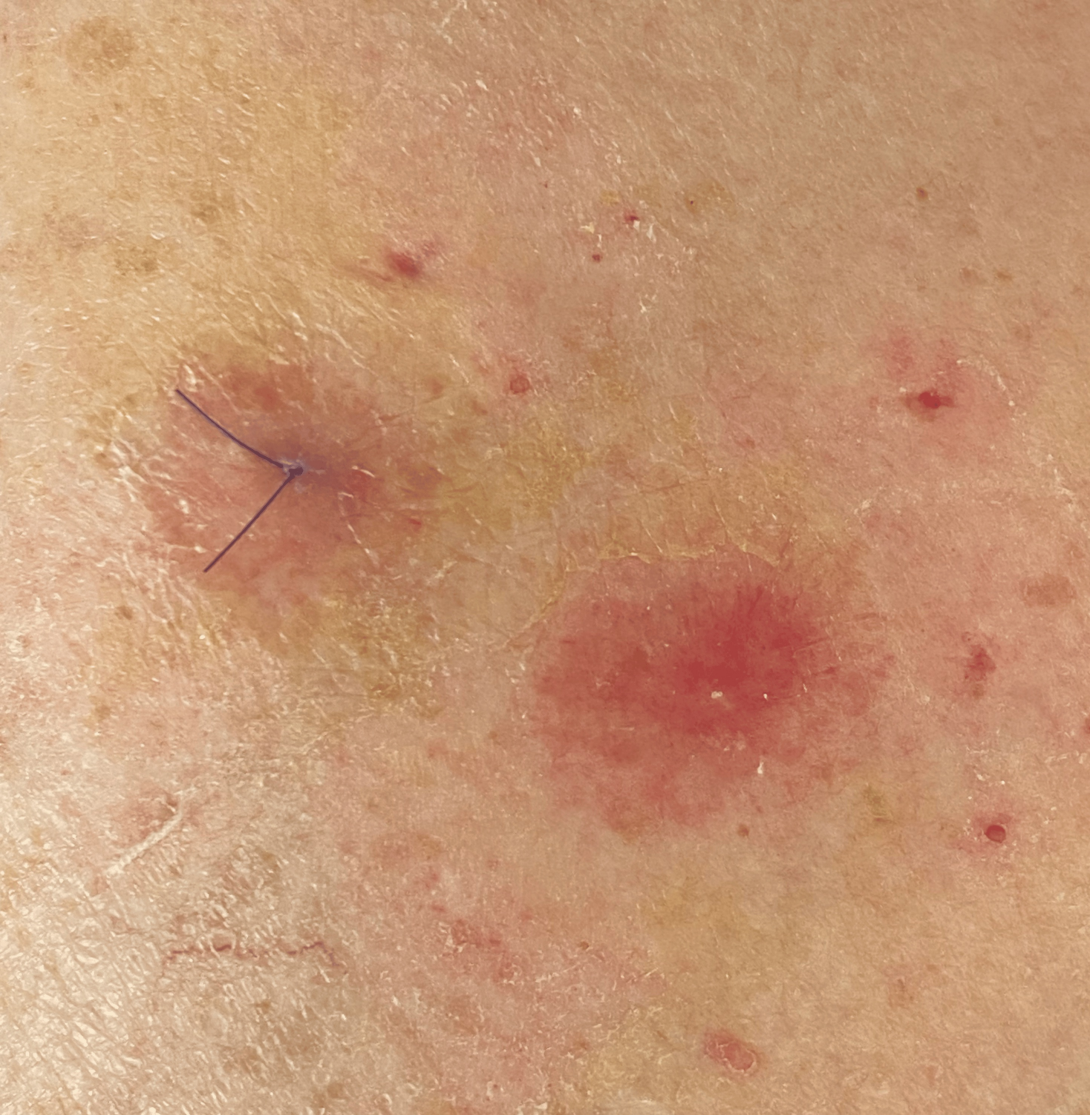
Two-month follow-up clinical aspect Partial regression of the initial lesion (left side of image, near suture), and appearance of a new similar lesion (right side of image).

Given the underlying neoplastic and immunocompromised context, actinic keratosis, squamous cell carcinoma, and achromic melanoma were considered as potential diagnoses. Biological tests were unremarkable. A skin biopsy confirmed the diagnosis of SLPK (Figure 3). The patient declined cryotherapy, and partial regression of the lesion was noted in the subsequent weeks. Most of the lesions had completely disappeared by the two-month follow-up visit, with the appearance of a similar lesion nearby (Figure 4).

## Discussion

SLPK was first described as a solitary form of lichen planus and later redefined as a LPLK. The pathogenesis of SLPK is thought to be driven by chronic inflammation, resulting in the regression of a pre-existing precursor lesion, such as seborrheic keratosis, actinic keratosis, or lentigo. The precise cause of the inflammation remains unknown [2,3].

The clinical diagnosis of SLPK is often challenging. It most commonly presents as a solitary lesion occurring after the age of 30, with a predilection for women and non-photo-exposed areas, including the back, parasternal area, upper and lower limbs, axilla, and neck [3], as observed in the cases of our two patients. SLPKs demonstrate considerable clinical variability: they may be raised or flat, pigmented or erythematous, ulcerated or scaly [4]. Diffuse LPLK can also be observed as an adverse reaction associated with chemotherapy or immunotherapy. To date, no studies have reported LPLK as a side effect of bortezomib; however, a potential association cannot be excluded and warrants further investigation [5,6].

Dermoscopy of SLPK does not present any pathognomonic findings [5]. They are frequently misdiagnosed as melanoma and non-melanoma skin cancers, such as Bowen’s disease and basal cell carcinoma (BCC). Indeed, SLPKs and BCCs share multiple dermoscopic features, including telangiectasias and milky red-white areas. While some dermoscopic features of SLPKs overlap with those of BCC, other characteristics are more specific to BCC, such as arborizing vessels, maple leaf-like areas, spoke-wheel areas, and blue ovoid nests [7,8]. When BCC lacks its specific dermoscopic characteristics, distinguishing it from SLPK becomes more challenging [7].

Nonetheless, certain features, particularly the presence of scales and orange areas, have been observed more frequently in SLPK and may provide helpful diagnostic clues [7,8]. Studies suggest that the orange color may serve as a diagnostic clue, even though it is non-specific and also seen in other conditions such as granulomatous diseases and xanthogranuloma [4,9,10]. In our case, the correlation between clinical and dermoscopic findings, namely the presence of scales and orange areas in non-photo-exposed regions, combined with spontaneous regression, supported the diagnosis.

Histopathological examination confirms the diagnosis, revealing irregular acanthosis with varying degrees of hyperkeratosis. The inflammatory infiltrate in the papillary dermis primarily consists of lymphocytes and lymphohistiocytes, without polymorphonuclear cells. Civatte bodies, pigment incontinence, and melanophagia are commonly observed [2-4,8].

The lesion typically regresses spontaneously [11,12]. Treatment, when performed, is conservative, including cryotherapy, curettage, electrocoagulation, or surgery. No recurrence or carcinomatous transformation has been reported.

## Conclusions

We believe that dermatologists must be familiar with the clinical subtypes and dermoscopic features of SLPK, as it is a completely benign cutaneous lesion. Given its wide clinical and dermoscopic variability and its frequent resemblance to malignant lesions such as BCC or melanoma, a thorough understanding of its presentation is critical. Recognition of dermoscopic clues, such as scaling and orange areas in non-sun-exposed locations, coupled with awareness of the potential for spontaneous regression, can guide clinicians toward a more accurate diagnosis. This knowledge is essential to minimize the number of patients undergoing unnecessary extensive surgical excisions.
